# A Multimedia Interactive Education System for Prostate Cancer Patients: Development and Preliminary Evaluation

**DOI:** 10.2196/jmir.6.1.e3

**Published:** 2004-01-21

**Authors:** Michael A Diefenbach, Brian P Butz

**Affiliations:** ^1^Fox Chase Cancer CenterDivision of Population SciencePhiladelphia PAUSA; ^2^Temple UniversityCollege of Electrical EngineeringPhiladelphia PAUSA

**Keywords:** Multimedia software, multimedia, software, prostate cancer, patient education, treatment decision making, treatment, decision making

## Abstract

**Background:**

A cancer diagnosis is highly distressing. Yet, to make informed treatment choices patients have to learn complicated disease and treatment information that is often fraught with medical and statistical terminology. Thus, patients need accurate and easy-to-understand information.

**Objective:**

To introduce the development and preliminary evaluation through focus groups of a novel highly-interactive multimedia-education software program for patients diagnosed with localized prostate cancer.

**Methods:**

The prostate interactive education system uses the metaphor of rooms in a virtual health center (ie, reception area, a library, physician offices, group meeting room) to organize information. Text information contained in the library is tailored to a person's information-seeking preference (ie, high versus low information seeker). We conducted a preliminary evaluation through 5 separate focus groups with prostate cancer survivors (N = 18) and their spouses (N = 15).

**Results:**

Focus group results point to the timeliness and high acceptability of the software among the target audience. Results also underscore the importance of a guide or tutor who assists in navigating the program and who responds to queries to facilitate information retrieval.

**Conclusions:**

Focus groups have established the validity of our approach and point to new directions to further enhance the user interface.

## Introduction

There is hardly a more-distressing health event in a person's life than receiving a cancer diagnosis. Such a diagnosis often has been compared with a "blow to the head," or the feeling that the "world has changed forever." Often, intense feelings of anxiety and fear about oneself and the future of family members are triggered by a cancer diagnosis. Yet, to make informed decisions about their health care during this time of high distress, patients and their family members need to process complicated medical information that is fraught with medical and probabilistic terms.

This is particularly true for men diagnosed with early-stage prostate cancer (ie, tumors confined to the prostate without involvement of regional lymph nodes or distant metastasis). Although prostate cancer is a highly-treatable disease with an average survival rate of 97% over 5 years [1], there is no one standard treatment that is appropriate for every patient [2,3]. Patients usually have the choice between surgical removal of the prostate (ie, prostatectomy), external beam radiation, or implantation of radioactive seeds. Watchful waiting, or close monitoring of disease progression through frequent prostate specific antigen (PSA) levels and digital rectal examinations (DREs), is sometimes an option. In addition, patients' treatment decision making is further complicated by the tendency of physicians to recommend treatment options that represent their medical specialty [4]. Thus, newly-diagnosed prostate cancer patients not only have to cope with the emotional impact of the diagnosis, but also have to make sense of complicated medical-treatment information, resolve often-times diverging medical opinions about how to best treat the disease, as well as to anticipate potential unpleasant treatment-related side-effects (ie, incontinence and erectile dysfunction [5]).

In recognition of these unique patient-information needs the primary aim of this paper is to describe the development and preliminary evaluation of a novel and innovative computer-based multimedia prostate interactive education system (PIES) and treatment-decision tool. We will begin with a brief overview of the most-common treatment options for prostate cancer and their likely consequences for quality of life. Second, we will describe how patients' information-seeking behavior has changed with the development of personal computers and the Internet. Third, we will place the development of PIES in the larger context of computer-aided learning. Fourth, we provide a detailed description of PIES and a report of its preliminary evaluation through focus groups. The paper closes with recommendations for further developments of computer-based education systems and decision tools.

### Computer-Aided Learning

Since the invention of the electronic computer there have been 2 divergent opinions as to how this device should be used. One group saw the computer's principal use as a number manipulator, an extensive, ultrafast, and accurate calculator. Another group envisioned the computer as a symbol manipulator that might be taught to use logic and make decisions in a human fashion. The symbol-manipulation group eventually founded the discipline called artificial intelligence (AI). As time passed, artificial intelligence spawned many subdisciplines such as robotics, natural language recognition, and expert systems. The driving force behind expert-system development was the attempt to program a computer so it could "think" and make human-like decisions. A generally-accepted definition of an expert system is one that performs at or near the level of a human expert in a particular field of endeavor [6].

The first large expert system to perform at the level of a human expert was developed for use in the health care field [7]. This system, called MYCIN, was developed at Stanford University in the mid 1970s. The MYCIN program was designed to provide physicians with advice about bacteremia and meningitis; it emulated the expertise from a physician specializing in these infections. MYCIN asks the physician, who is requesting its advice, several questions. Additionally, it requires personal data about the patient and will ask for results of laboratory tests when it needs this information to aid in its analysis process. After analyzing patient information and data, MYCIN makes a diagnosis as well as a drug-therapy recommendation. MYCIN also has the ability to explain how it arrived at its diagnosis [8].

The architecture of the MYCIN program consists of 2 major, distinct elements: (1) a knowledge base and (2) an inference engine. The knowledge base contains the facts and heuristics necessary to derive medical analyses of bacteremia and meningitis infections. The inference engine contains the inference mechanisms and control strategies employed by MYCIN to derive the diagnosis and treatment recommendation. It soon became clear that this architecture was not just specific to MYCIN but could be used as a template or "shell" for encoding and applying expertise in any field.

### Computer-Aided Learning for Educational Purposes

One of the most interesting applications of expert system technology is for education. One goal among some artificial-intelligence researchers has been to develop a computerized tutor that performs at the level of an excellent human tutor. The resulting computer program that achieves this objective would be called an intelligent tutoring system (ITS). The effort to create truly-intelligent computer-based tutors has been underway for many decades [9]. Ultimately intelligent tutoring systems attempt to simulate the behavior of an intelligent human tutor in addition to acting as a domain expert. The characteristics of an intelligent tutoring system include the ability [10,11] to:

1. teach a given subject
2. detect student errors
3. analyze where and how the student made an error
4. correct flaws in the student's logic
5. clear up any misgivings or misunderstandings the student may have about the material.

Although the development of expert systems for training has been moderately successful and other kinds of expert systems, such as income-tax preparation assistants, have been extremely successful commercially, intelligent-tutoring-system development did not advance significantly for several years after its initial efforts. It is within the last decade that prospects for intelligent tutoring systems have shown promise. Advancements in hardware, operating systems, and interactive-multimedia development tools have created a software environment that makes real intelligent tutoring systems a possibility [12- 14].

Interactive multimedia software is already playing a key and unique role in the educational process. It is used as a stand-alone educational module that is intended to enable an interested individual to learn about a particular topic or subject [15]. It has been used as a supplement to classroom presentations [16,17] and laboratories [18,19], and it is used as a dynamic textbook [20]. Recently, interactive multimedia have been integrated with expert-system technology, producing highly-interactive intelligent tutoring systems [21- 25]. These intelligent tutoring systems "learn" about the individual student and can tailor the material to meet the needs and the learning styles appropriate to the individual student.

### Computer-Aided Learning for Health Care

One of the first successful large-scale expert systems was developed for diagnosis and treatment of certain infections [26]. The principles of this approach were subsequently applied to include the application of artificial intelligence techniques to medical diagnosis (for example, [27- 32]). Artificial intelligence research within medicine is very active today although attempts to develop physician-like diagnostic systems have waned. Instead, these systems are now geared to assist students and physicians to develop and hone diagnostic skills.

Because enthusiasm for medical diagnosis systems that would "assist" physicians and might even dispute their diagnoses was tepid, the development of systems designed for practitioner education has been advanced. Consequently, considerable effort has been made to produce software systems that could simulate medical situations and include a computerized mentor that would assist learners in their decision-making process. The rapid development of computer hardware has eliminated many of the early problems encountered in developing medical-training expert systems. Today's graphical user interfaces coupled with developments in various video and audio digital circuit boards have encouraged the development and use of multimedia software. Multimedia is usually defined as "the use of a computer to present and combine text, graphics, audio and video with links and tools that allow the user to navigate, interact, create, and communicate" [26]. Multimedia programs have been written to help educate medical practitioners in almost every aspect of health delivery. Systems exist to train and educate individuals in the principles of: laparoscopic procedures and epilepsy diagnosis and treatment [33], ureterorenoscopy [34], hysteroscopy [35], dermatology [36], diabetes management [37], communicable and rare diseases [11], red cell antibody identification [38], cervical cytology [39], psychotherapy [40], and many other health-related areas.

Besides educating practitioners, progress has been made recently in developing interactive multimedia software products for patient use and education. Agre et al, reporting on a project that develops a CD-ROM for cancer-related patient education [41] notes that CD-ROMs are more valuable for learning than booklets or videotapes. The study finds that CD-ROM technology allows for greater depth in content and has the ability to satisfy a broad range of educational needs. Guendelman et al [42] have used a computer-based self-management program called Health Buddy to enable children to assess and monitor their asthma symptoms and quality of life as well as to transmit this information to health care providers. Using a randomized control trial, 66 individuals were placed in the intervention group while 68 were in the control group. The study found that compared with an asthma diary, monitoring asthma symptoms and functional status with the Health Buddy increases self-management skills and improves asthma outcomes. Another recently-developed CD-ROM focused on the education of children with leukemia (ages 4-11) and their families about the disease and its treatment [43].

Multimedia has also been used, with some success, for educational intervention for low-education, low-income Latinas [44]. At the conclusion of the study, it was found that about 40% of the women that had scheduled or received mammography had attributed their decision to the intervention. Interactive multimedia has been used to deliver information: about self-medication [45], for patient colonoscopy education [46], for genital herpes education [47], for personal care of diabetes [48], for skin cancer prevention [49], for nutrition screening and counseling [50], for hypertensive patients [51], and for numerous other diseases or treatments.

Gustafson et al have developed a computer-based health information and support system [52,53]. This Comprehensive Health Enhancement Support System (CHESS) is a Web-based support system that includes modular programs on breast cancer, AIDS/HIV infection, sexual assault, alcoholism, and academic crisis. For example, the breast cancer module provides disease information, a treatment decision aid, an opportunity to contact health care providers via e-mail, testimonials from patients, and a patient forum to exchange information and to solicit social support. Evaluation studies have confirmed that the CHESS system is user-friendly and well accepted by patients of diverse demographic background [54]. The use of the CHESS system has also been linked to improvements of quality of life, and increased participation in health care among HIV-positive patients [54]. Despite its sophistication, the CHESS system lacks the capacity to present information in a targeted and tailored manner based on specific patient characteristics.

### Computer-Aided Tailored Patient Education

A decade ago researchers [10] noted that computer software has the potential to elicit information from patients and to use this information to tailor patient education. Because the literature is not always consistent in the use of the terms *targeted* and *tailored* with respect to health communications, a recent article has suggested clarification and standardization in terminology [55]. The authors suggested that health communications be labeled as targeted if they are directed towards a particular subgroup of the population, and to use the term tailored if the information is designed to address individual characteristics of a person. Both targeting and tailoring depend on the assessment of group or individual characteristics that have been derived from prior data collections [55].

The goal of targeting and tailoring health communications is to increase the personal relevance of the message to the individual. Research has demonstrated that messages that are relevant to the person are better understood and better remembered [56,57]. In addition, personally-relevant messages are also more likely to lead to behavior change [58]. Thus, it is generally expected that an individual will react to tailored messages with increased attention, improved understanding, and an increased tendency for behavior change [54,59].

## Methods

### Overview of the Prostate Interactive Education System (PIES)

**Figure 1:**
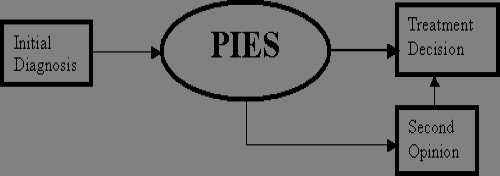
Role of prostate interactive education system (PIES) in the treatment decision process

The prostate interactive education system (PIES) is an interactive multimedia expert system that is being developed to help patients who have been diagnosed with early-stage prostate cancer. PIES provides the patient with a multitude of treatment information and encourages patients to obtain the kind of information that they desire about the disease. PIES is envisioned as an important step in the treatment-decision process. The software will enable a patient to follow a logical decision process and obtain the information he needs at his current state of decision-making. The software will prepare a factual report for a psychologist who will meet with the patient following his interaction with the software. The temporal placement of the software is shown in Figure 1. Once the patient is diagnosed with prostate cancer, he is offered the PIES software package to assist him in making a treatment decision. PIES provides additional information and does not preclude a second opinion, as shown in Figure 1. In fact, the patient may receive a second opinion before using PIES.

The purpose of the PIES software is threefold:

1. It allows a patient to obtain the information he wants in a quiet environment. The patient interacts at his own speed. Questions can be asked in any order. Multiple physicians are consulted without any implied obligation on the patient's part. A patient may consult with a surgeon and a radiologist. Consequently, the patient can gather information in a nonthreatening and unpressured environment.
2. It allows the patient to access the experiences of other patients. An extensive library of interactive videos of others' experiences will be available to the patient. The patient can choose to hear from videotaped actual patients who are in the same age group, of the same race, and who had a particular treatment.
3. It provides an expert system that will be operating in the background. The expert system will analyze what the patient is doing and determine if the patient is getting enough information to make an informed decision. The expert system will not make any decision for the patient. It will only follow and evaluate the completeness of the process. If the expert system becomes concerned that the patient is not being thorough in his examination of alternatives, it will make suggestions to view information that has not been accessed and about where the patient might find it. The patient can follow or ignore the expert system's suggestions.

PIES is not a diagnosis tool. Nor does it give advice in the form of "this is what you should do." Instead, it allows patients to ask questions and get information by interacting with a multimedia computer program. The multimedia program allows a patient to visit a library to get up-to-date information about prostate cancer and its treatment. The patient will be able to interact with virtual physicians who can answer specific questions that the patient may have. Various physicians (eg, surgeons, radiologists) can be queried by the patient. The physicians "consult" with the patient through digital video sequences as well as through interactive multimedia question-and-answer sessions. The patient will also be able to "visit" a virtual support room where he will have the chance to listen to other patients talking about the disease and how they determined their own course of treatment. Support group members also discuss dealing with the side effects of their treatment. The patient will also be able to consult with a virtual sex therapist who will discuss methods and assistive technologies that will enable them to continue sexual activities. Finally, each patient will be provided with a decision aid that will assist him in treatment decision making.

**Figure 2:**
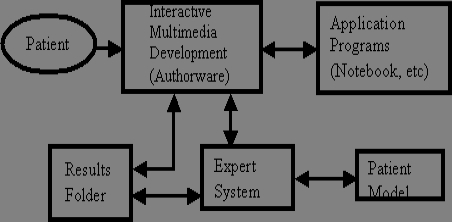
The PIES architecture

An overview of the PIES architecture is shown in Figure 2. The patient interacts with the CD-ROM based system on a personal computer. The system is CD-ROM based but could be made available over the Internet. A CD-ROM delivery system was chosen so that those patients without a high-speed connection to the Internet would not experience delays caused by downloading videos. The information is developed and presented using Macromedia's Authorware [60]. Authorware is able to communicate with other software and can transmit information in real time or near real time. Authorware will allow the patient to have access to other programs such as a notebook to record information, and various applicable medical-applications software.

The patient model, described later, is a computer characterization of the patient; it is changed dynamically by the expert system. In turn, the expert system, also described later, analyzes how the patient interacts with the software and reports this in the results folder. The results folder contains information that the expert system will use to generate a report to the psychologist.

The paradigm used in PIES is that of a patient entering a health center. When the patient enters PIES, he is greeted by an information specialist. The information specialist welcomes the patient and shows him around the PIES Health Center. The Health Center consists of a reception area, a library, and a group meeting room on the first floor, and physician offices on the second and third floors, connected through an elevator. Each room is interactive and the patient is shown how to use the room's facilities when he first chooses to enter the room. After showing the patient the Health Center layout, the information specialist asks the patient to complete a questionnaire. The questionnaire requests data that the expert system will need to tailor the Health Center for his needs.

After completing the questionnaire, the patient is free to visit any room of his choosing.

### Elements of PIES

#### The Library

**Figure 3:**
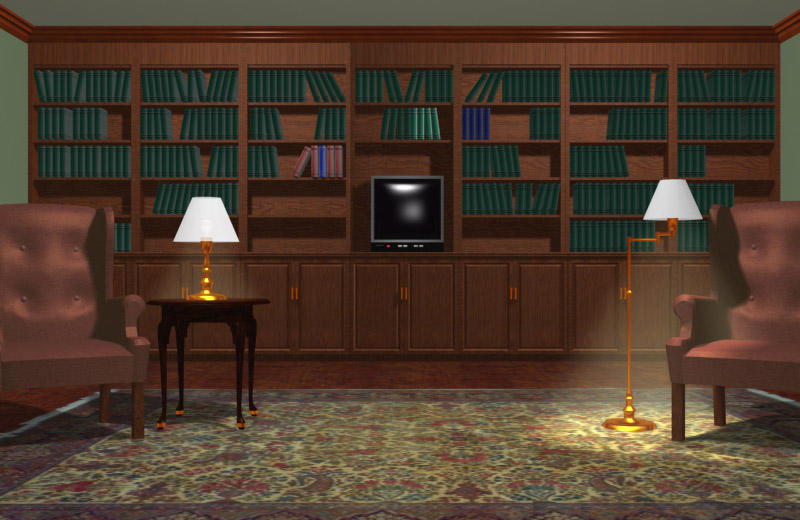
The PIES library

**Figure 4:**
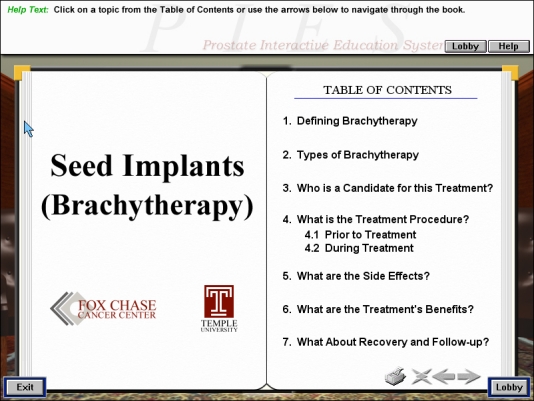
Table of contents page for book on brachytherapy

If the patient would like information, he is referred to the library. To get to the library, or any other room, the patient would click with the mouse on the door that leads to the desired area. The library is a highly-interactive area (see Figure 3) in which the patient may obtain, and interact with, educational material and other information. The library consists of a wall of shelves with many books to choose from. It also has a TV through which videos can be accessed. The books are sorted in alphabetical order; by "rolling over" a book with a mouse, its title is revealed. For example, a book entitled Brachytherapy (see Figure 4) contains information about radioactive-seed implant treatment. A chapter gives an overview of brachytherapy; another chapter focuses on side effects, while another one describes the rationale behind a particular treatment regimen. Other books available contain information about psychosocial functioning, such as how to deal with impotence and incontinence, the use of alternative medicine, clinical trials, and the impact of prostate cancer on the family. The video section contains short videos (up to 5 minutes) that show facilities (eg, a surgical suite) and describe specific treatments. In sum, the library is a place that has a comprehensive collection of disease- and treatment-related materials that not only address the momentary concerns of the patient, but also assist in the preparation for future prostate-cancer and treatment-related consequences.

#### Sources of Information

The identification and development of the information contained in PIES is a multi-step process. A starting point for compiling information is a review of current information on prostate cancer treatment (eg, the PDQ [61]), that is approved by the National Cancer Institute [62] and the Cancer Information Service [63]. Regular Medline [64] searches supplement this information and ensure that it stays current. Second, the research team summarizes this information and identifies and develops appropriate visual materials. Third, medical expert consultants vet all materials for accuracy. Fourth, a health educator and a cancer-information specialist adopt the information to a 6th- to 7th-grade reading level. Last, information is then adopted for high and low information seekers.

#### Interacting with Physicians

**Figure 5:**
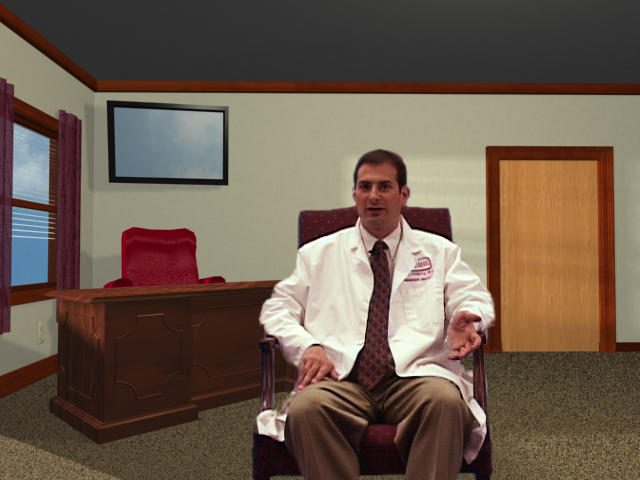
Meeting with a physician

The physician offices are on the second and third floor of the virtual health center. The physician offices are reached through an elevator, which contains a large sign that allows the user to click on the desired office. Experts in a treatment area (ie, surgeons, radiation oncologists, a physician specializing in brachytherapy) are available to provide information about different treatment modalities. After entering the office (see Figure 5), the patient interacts with the physician by selecting different video clips from a pull-down menu. Each video clip answers specific questions about the treatment, providing anatomical and technical information. In addition, physicians discuss issues such as who is a candidate for this treatment, the likelihood of side effects, success rates, recovery time, and expected quality of life.

#### The Group Meeting Room

**Figure 6:**
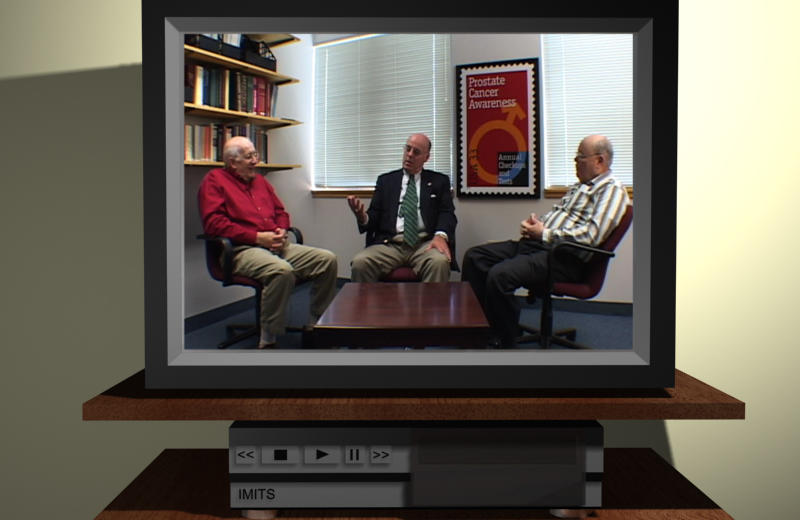
Viewing a support-group session

The patient may choose to participate in a group meeting of prostate cancer survivors. The members of these groups have experienced the range of treatment options. The patient may listen to the group members discuss with one another aspects of their treatment decision-making such as how they found out that they had prostate cancer, how they felt when they found they had the disease, and how they chose a treatment. Figure 6 shows members of the group discussing a wide-ranging array of topics that include sexual and incontinence problems, issues with intimacy, the effect of the disease on the partner, the influence of the spouse on treatment decision-making, experience with different treatments, and the use of alternative therapies.

#### The Notebook

If the participant wants to take notes or jot down a comment during his exploration of the Health Center, he can call up an electronic notebook, which is available from any room. The notebook will also be helpful to jot down notes when visiting the doctor-patient module. These notes might be helpful to the patient when he consults with physicians about prostate cancer. All notes will be incorporated in the final printed report that will be generated upon exiting the program.

#### Exiting the Software

When participants decide to exit, the software calculates which information had been accessed and offers information that has not been viewed to patients. For example, if a patient only inquired about surgical treatment for prostate cancer, the program offers information on external beam radiation and brachytherapy for reviewing. Thus, the software will ensure that all patients have had a balanced amount of information. If the patient desires, he can complete a decision aid, designed to elicit his values and goals with regard to prostate cancer treatment [65] and thus assist him in making a treatment decision that is right for him. After completing the decision aid, the software will also ask the patient if he wants to return to PIES. If the answer is yes, the expert system will update the patient's results folder before the program is closed. When a participant re-enters the software he is placed in the reception area to decide which part of the health center he wishes to visit. If the participant does not intend to revisit the software, it updates the results folder, and then generates a report for the patient. This report contains a synopsis of accessed information, notes that were taken, areas the participant has not visited, and types of available information that he has not yet accessed.

### Tailoring to Information Seeking Within PIES

Improved communication between physicians and patients and improved information provision about disease and its treatment are important factors in aiding patients to make treatment decisions and to cope with their disease [21,66,67]. Although, patients are generally interested in obtaining information about their disease, not all patients desire the same level of information [68,69] and thus an individualized approach to information provision is more appropriate to meet a patients information needs [70,71].

Research has identified 2 main profiles of information processing: high monitoring (information seeking) and low monitoring (information distracting). When confronted with medical information, high monitoring individuals typically process information very attentively and amplify its threatening aspects. High monitors prefer more information about their condition and its treatment, are consequently better informed, yet express greater dissatisfaction about the amount of information they receive [68]. Overall, they demand more support and reassurance [68,72].

In contrast, low monitors demonstrate an opposite pattern; they are more likely to ignore, avoid, and minimize threatening cancer-related information that diverges from their belief that they will be fine [41,73- 76]. Consequently, low monitors prefer and are satisfied with less information about their condition, are less knowledgeable about their disease status, and tend to underestimate the risk from the disease threat.

The expert system determines if the patient is a low monitor (ie, low information seeker), one who wants only the important points of a treatment, or a high monitor (ie, high information seeker), one who wants considerable detail about a treatment therapy. The expert system directs the interactive multimedia program to provide the amount of information that the patient desires. The expert system realizes that a patient might want to be a high monitor in some areas and a low monitor in others. For example, a patient may be very interested in knowing all about the side effects of external beam radiation while wanting only the essentials about the procedure itself. To our knowledge, PIES is unique in that it allows dynamic tailoring. Consequently, as patients proceed through PIES, its expert system determines how much information patients desire about a topic and provides it.

### Survey to Establish Computer Literacy Among Target Audience

Before developing PIES we conducted a survey among the target audience (ie, men diagnosed with localized prostate cancer) to establish familiarity with computers. We mailed a brief questionnaire to 675 participants of an ongoing longitudinal study of prostate-cancer treatment decision making and quality of life [5].

### Focus Groups to Evaluate PIES

We developed a demonstration CD of PIES to showcase its features to several focus groups. The first 3 focus groups consisted of 18 prostate cancer survivors. Men were recruited from the same ongoing longitudinal study mentioned above [5].

## Results

### Computer Literacy Among Target Audience

Of the 675 survey questionnaires mailed, 473 were returned (70% return rate). Demographic characteristics are listed in Table 1.

**Table 1:** Demographic characteristics of PIES users

**Characteristic**		**Mean (SD)**	**Percentage of Sample**
Age in Years		66 (8)	
	35-45		0.2
	46-55		10
	56-65		34
	66-75		47.8
	> 75		8
Ethnicity			
	Caucasian		92
	African American		6
	Hispanic		1
	Asian/Pacific Islander		1
Education			
	Completed grade school		7
	Completed high school		47
	Completed college		23
	Post-graduate degree		23
Marital Status			
	Married		85
	Single/separated/widowed		15
Employment			
	Retired		60
	Full- and half-time employed		40

The majority (65%) of participants reported they have a PC at home, and an additional 7% indicated they had access to a PC at a public place, such as at work or at the library. Access to a computer came with access to the Internet for 70% of the respondents. When asked to rate their experience in using a computer, 80% of patients indicated they are familiar with computers. They rated their expertise from slightly experienced (39%), moderately experienced (18%), very experienced (11%), to expert (2%). Three quarters (76%) further indicated that they had access to a family member or a friend to help them with potential computer problems. When asked about any physical problems that might prevent them from using a computer, 89% of patients reported not having any eye problems that might interfere with using a computer, and 83% indicated they have no difficulty at all in operating a pointing device such as a mouse (10% indicated having "a little problem" with a mouse). Patients who have a computer at home spend an average of 3 hours (SD = 1.5 hours) per week using it. About 20% spend more than 2 hours per day on the computer. Thus, our target audience for PIES is experienced in using a computer.

Of the 18 men, 7 (39%) were African American. We oversampled African American men into the focus groups, in part because, prostate cancer is particular prevalent among African Americans and thus the use of the program might be specifically relevant to this group. Men were on average 67 years old and had at least a high-school education (33%), a large majority was married (83%), and had completed treatment (83%). External beam radiation was chosen by 72%, 22% chose surgery, and 6% chose brachytherapy. The remaining 2 focus groups were held with spouses of prostate cancer survivors (N = 15). The women were on average 60 years old and 50% had a college or postgraduate degree. All focus group participants received $40 as a token of appreciation.

### Focus Groups' Evaluation of PIES

Overall feedback on the demonstration CD was extremely positive. All ratings were performed on a 5-point scale, with higher scores indicating higher levels of interest. Patients and spouses uniformly stated that they were "very much" interested in the software (mean = 4.71; SD = 0.59; range, 3-5), and that it was "very" useful (mean = 4.71; SD = 0.47; range, 4-5). When asked whether they would prefer the software in lieu of traditional information sources, such as books or booklets, 83% stated a clear preference for the software program over print material, and 100% would prefer the program in addition to print material. On average, patients indicated that they would spend between 1 and 2 hours with the program and were willing to pay an average of $50 for it, if it were commercially available. A large majority of patients (82%) indicated that they had access to a personal computer and that they frequently use it (41% everyday, 29% 3-4 times a week, 18% 1-2 times a week); 94% had access to the Internet. In general, patients use their computer for e-mail (71%), and to obtain general (77%) and health (71%) information. Almost 60% have used the Internet to obtain prostate cancer information.

#### Concept of a Virtual Health Center With Rooms or Offices

Transcripts of the participants' comments revealed that participant's liked the concept of a virtual health center. Uniformly, the idea of going to rooms or offices to obtain information was found intuitive and appealing. Further, participants appreciated the variety of information that could be found in the program. The following summarizes comments by focus group members, organized by rooms.

##### Introduction

Men and spouses found the Introduction to PIES easy to follow and indicated that it gave an excellent overview of the program. They particularly liked the possibility of accessing information in any order they liked and the program's capability of tailoring the information to their information-seeking needs. Participants also mentioned that they value an interface that mimics an interaction with a human. Some men suggested the inclusion of a guide who follows them around in the health center and who could be asked questions.

##### Physician's Office

The large majority of men and spouses (90% for each) indicated that they would visit the physician's offices first, before going to any other room. Participants liked the opportunity to type in a question, which will prompt the program to retrieve the appropriate video with the physician answering the questions. However, a majority of patients also requested an overview of available physician answers. As one man stated: "After a diagnosis I didn't know what type of questions to ask. An index of available information from the physician would be very helpful." Some men also didn't like the physician sitting behind a desk, which increased the perceived distance between patient and physician. The current version of PIES places the physician in a comfortable chair in front of the desk.

##### Library

Participants loved the layout of the library and found the idea of providing information in book form very appealing. They particularly appreciated the combination of written text with illustrations and short video clips. Some men voiced interest in watching video clips of surgical or seed-implantation procedures. Others, in contrast, indicated that they would not be interested in such a level of detail, providing unwitting support for our plans to tailor information to their monitoring preference. Both patients and spouses liked that some medical terms were hyperlinked to the Glossary, which provided short one-sentence explanations of the term.

##### Support Group

In general, men showed great interest in watching video clips of prostate cancer survivors sharing their experience. In the initial format there were 3 men sitting behind a table answering questions that were keyed in by the patient. While about half of the patients appreciated the opportunity to interact with each man directly in the support group, the other half was interested in watching the men exchanging their ideas. The current version of the software includes videotapes of men discussing certain topics, such as treatment decision-making, treatment experience, and post-treatment quality of life.

#### Topics Specifically Mentioned in Focus Groups With Spouses

Certain topics were specifically mentioned in the focus groups with spouses. Spouses advocated for a room that provided information specific to their information needs. Topics of interest were information about nutrition (eg, soy, lycopenes), emotional support (both resources for support, as well as learning from the experience of other spouses), as well as instrumental support, particularly with caregiving after treatment. Spouses were also interested to receive information about sexual issues as it pertains to intimacy, communicating to one's spouse about sexual issues, and the use of devices to assist a patient with erectile dysfunction.

### Summary

In summary, both men and spouses were enthusiastic about the program. They expressed great interest and regretted not having had such a tool when they had to make a treatment decision. When given the choice between traditional information sources such as books or pamphlets almost every participant indicated a preference for the CD-ROM. Of particular interest to patients were the physician's office and the support-group room, which offered patients expert as well as lay perspectives of prostate cancer treatments. Although men expressed proficiency with computers in general, it became clear that additional navigational and organizational help would enhance the usability of PIES.

## Discussion

In this paper we have introduced the development and preliminary evaluation of novel multimedia-education software for prostate cancer patients. The article started with a brief review of the major developments in computer-aided learning, particularly emphasizing research and development efforts in the area of providing (tailored and nontailored) health information and education. We then introduced the PIES architecture, described the visual interface, content development, and preliminary evaluation through focus groups.

### Plans for a Virtual Information Specialist and Interactive Tutor (VISIT)

Overall, the focus group members evaluated PIES very positively and provided important suggestions about potential changes and enhancements to the program. Based on these comments and in accordance with health-behavior and communication theories [7,77] we are planning to enhance the next version of the PIES software by including a virtual information specialist and interactive tutor (VISIT). This information specialist and tutor will be designed to further enhance the interactivity between user and software. Specifically, VISIT will proactively query the user about what kind of information he desires, inform him where he can obtain the information, and will offer to retrieve it for him. VISIT will further guide the user through the program by offering relevant information that had not been accessed, thus anticipating the user's informational needs. Furthermore, while offering treatment information that could be upsetting to some patients the program will also offer emotionally-reassuring and normalizing messages.

**Figure 7:**
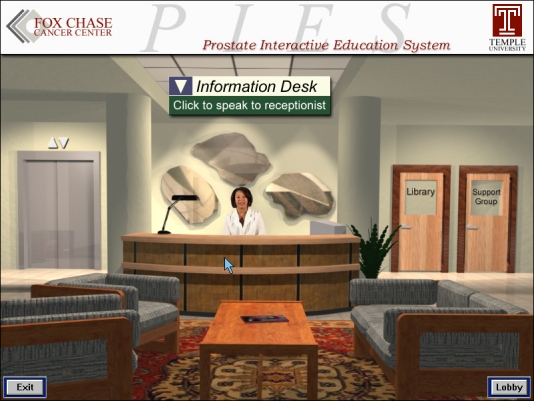
The virtual information specialist and interactive tutor (VISIT) at her desk

VISIT will be represented as a health care professional (see Figure 7) through video and still photos. Once the patient enters the health center, she will introduce herself and will give an overview of the center. She will offer suggestions on how to best use the program, depending on the user's needs. For example, if a patient is interested in hearing a physician's opinion, VISIT will offer to show him the way to the doctor's office. In contrast, if the patient is interested in learning about different treatment modalities in great detail, VISIT will accompany him into the library. Once there, VISIT will query the user about which treatment options he might want to learn about first and which aspects he is most interested. The corresponding information will then be presented.

#### Areas in Which VISIT Will Increase the Functionality of PIES

##### Provide Balanced Picture of Treatment Options

Most importantly, VISIT will ensure that the patient receives a balanced picture of all treatment options. Patients who are accessing information about only one treatment option will be queried by VISIT as to whether they are interested in receiving relevant information about other treatment modalities. VISIT will present the information or offer to take the patient to the appropriate room.

##### Increase Retention of Information and Provide Emotional Reassurance

VISIT is designed to increase the patient's retention of the presented information and to provide emotionally-reassuring and normalizing messages. In particular, after a module is completed VISIT will summarize the information that was given to the patient. In this context, VISIT will emphasize that prostate cancer is a common disease among men, that the tumor is slow growing and highly treatable. Furthermore, VISIT will offer information about support groups and additional information on the Internet.

##### Raise Patient's Perceived Self-Efficacy

Another area that will be addressed through VISIT is to raise the patient's perceived self-efficacy to deal with his condition. On the one hand, this will be achieved by providing information that is tailored to the patient's information-seeking needs. On the other hand, VISIT will provide concrete suggestions designed to enhance the patient's communication skills with his provider. VISIT will display sample interactions between patient and physician.

Short video clips depicting situations in which the patient is successful in requesting and obtaining information about his treatment options from the physician will represent these interactions. After watching these short video clips, VISIT will summarize the most important communication practices, including sample questions, that the patient can use to enhance his communication with his provider.

##### Suggest Completion of a Treatment Decision Aid

Finally, after the user has accessed a sufficient amount of information, VISIT will suggest the completion of a treatment decision aid. Completing a decision aid will be a 2-step process and follows the latest findings in theory and research [36].

In Step 1 the patient is asked to identify and rank his treatment-related values and goals. Specifically, he is asked to complete a table that will allow him to list attributes that are important for him (such as high rate of success, convenience, avoidance of pain). The patient can choose from a list of commonly-named treatment-related attributes as well as include and rank his own goals. When completed this table will consist of his own list ranked by importance of treatment-related goals.

In Step 2 the computer will carry over the ranked items into a larger table that consists of rows and columns. In column 1 the ranked items appear; the columns to the right represent the different treatment choices (eg, surgery, brachytherapy). Now the patient is asked to rate how well, in his mind, each treatment addresses his ranked attributes. Ratings will be done on a 5-point scale (1 = not at all, 5 = completely). If the patient feels that he has not enough information he can enter a question mark. This will serve as a reminder to either query PIES for further information on this issue or discuss this issue with his physician. The computer will automatically tally up the ratings and will summarize the results for him. A sample paragraph could read like this:


According to your ratings, it is very important to you to not experience urinary problems 
and to be able to maintain an active lifestyle. You also mentioned that you favor a high 
likelihood to cure the cancer, and are quite a bit worried about pain and recovery time. 
Your ratings suggest that you are leaning towards radiation therapy, specifically 
external radiation therapy. To learn more about this therapy please click here. You might 
also want to talk to a radiation oncologist about this treatment option.


After the user completes the decision aid, VISIT will ask whether the user would like to print any of the patient's notes before exiting the program.

### Limitations

Our preliminary evaluation has some limitations. First, focus group members evaluated a demonstration CD, designed to elicit input about the metaphor, its presentation, and possible content. These individuals had successfully completed treatment and thus their information needs were not as immediate as those of just-diagnosed patients. Yet, a number of survivors and spouses mentioned that they wished they had PIES while they were learning about prostate cancer. It should also be pointed out that PIES is intended to serve as an additional patient-education tool. It is not designed to substitute for personal consultations with a physician. Indeed numerous statements will be made to encourage the patient to discuss specific individual medical issues with a health care provider.

### Next Step

The next step in the evaluation of PIES will involve extensive usability testing with a patient sample. We will use the methodology and procedures set forth by the National Cancer Institute's own usability program (Usability.gov, [78]). Patients will be asked to talk aloud while exploring an alpha version of PIES, detailing what type of information they are looking for, where they think they can find it, and their reactions to it. Based on current usability standards we estimate that a small number of users (ie, approximately 5 patients) is sufficient to detect the majority of bugs. All suggestions from the usability version will be incorporated into a beta version of the program. This version will then be evaluated for efficacy in a randomized controlled trial, in which PIES will be evaluated against standard care (ie, information provided through brochures and pamphlets).

### Conclusions

In this paper we have introduced a novel interactive multimedia system for prostate cancer education and treatment decision making. PIES is based on current psychological theory incorporating tailoring to information-seeking preferences with the latest software technology. The program is highly interactive, combining user input with animation, video, text, and still photos. Preliminary evaluation through focus groups has established the validity of our approach and has pointed to new directions to further enhance the user interface through the development of a virtual information specialist and interactive tutor.

## Abbreviations

PIES: prostate interactive education system
VISIT: virtual information specialist and interactive tutor

## Multimedia Appendix

Screenshots of PIES (Prostate Interactive Education System):
