# Supplementary material for: A Multimedia Interactive Education System for Prostate Cancer Patients: Development and Preliminary Evaluation
Source: J Med Internet Res. 2004 Jan 21;6(1):e3. doi: 10.2196/jmir.6.1.e3 (PMC1550590; doi:10.2196/jmir.6.1.e3)
Supplement: Supplementary file 1 [file jmir_v6i1e3_app1.ppt]

## Slide 1
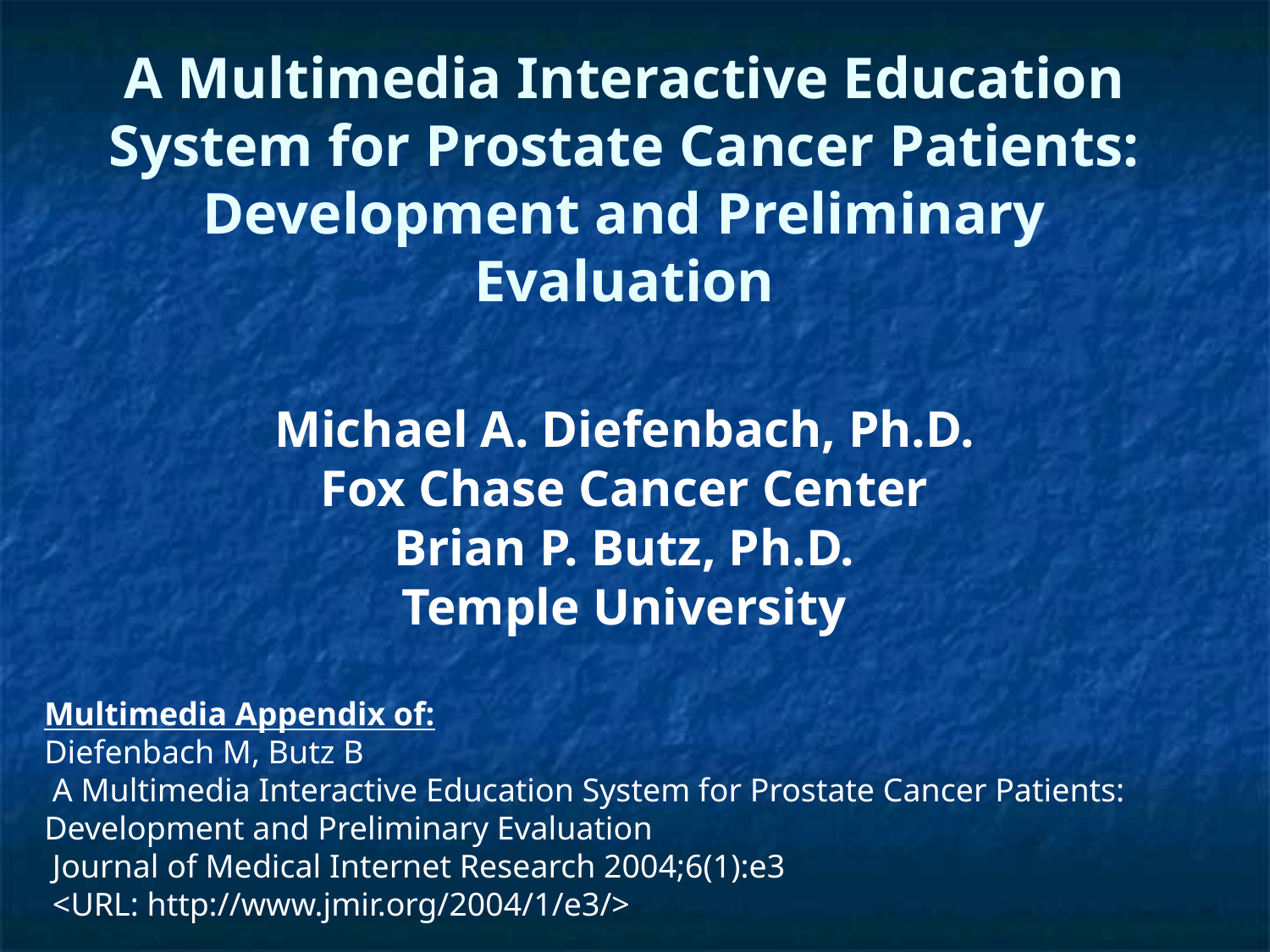

# A Multimedia Interactive Education System for Prostate Cancer Patients: Development and Preliminary Evaluation
Michael A. Diefenbach, Ph.D.
Fox Chase Cancer Center
Brian P. Butz, Ph.D.
Temple University
Multimedia Appendix of:
Diefenbach M, Butz B
 A Multimedia Interactive Education System for Prostate Cancer Patients: Development and Preliminary Evaluation
 Journal of Medical Internet Research 2004;6(1):e3
 <URL: http://www.jmir.org/2004/1/e3/>

## Slide 2
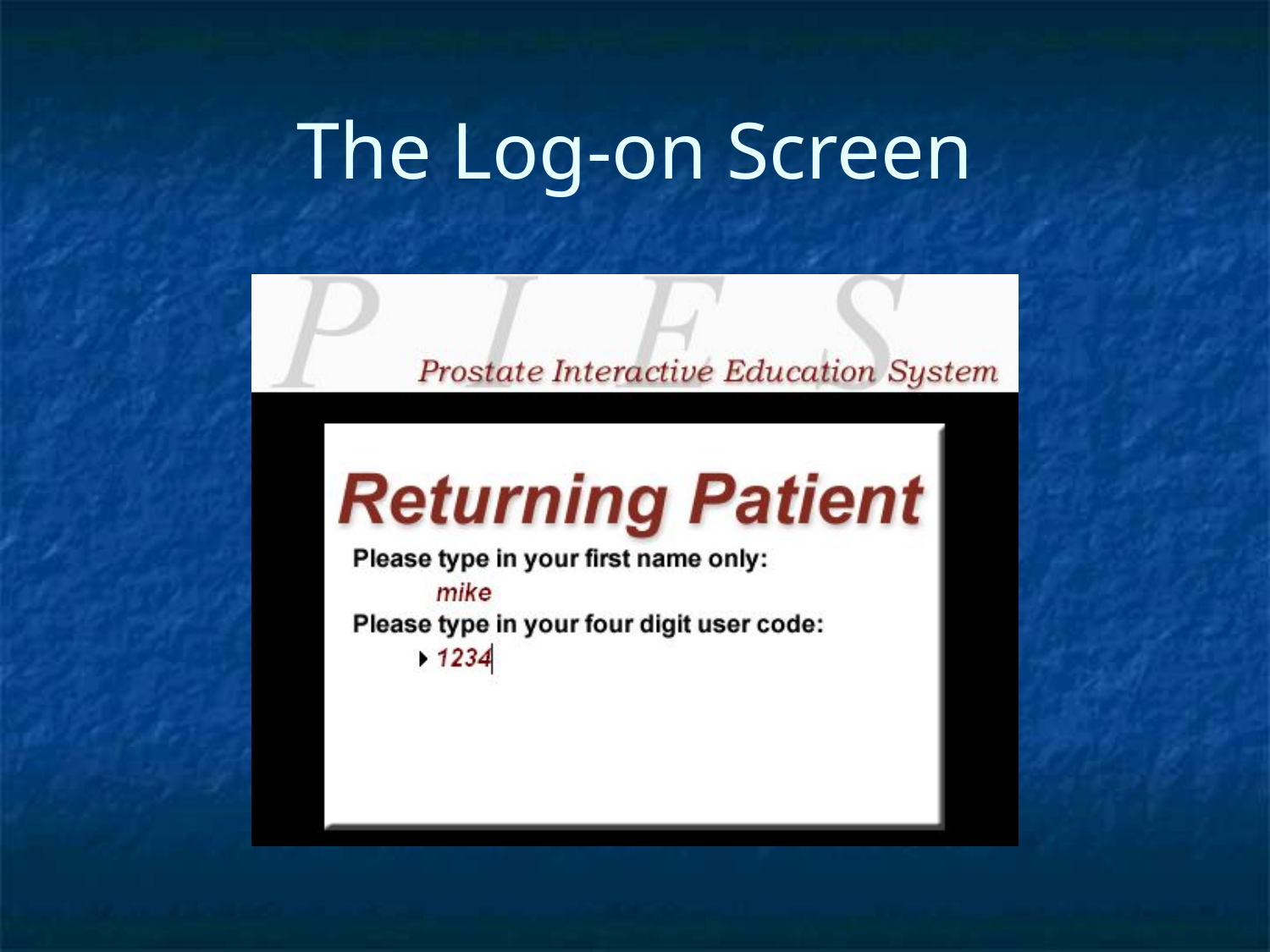

# The Log-on Screen

## Slide 3
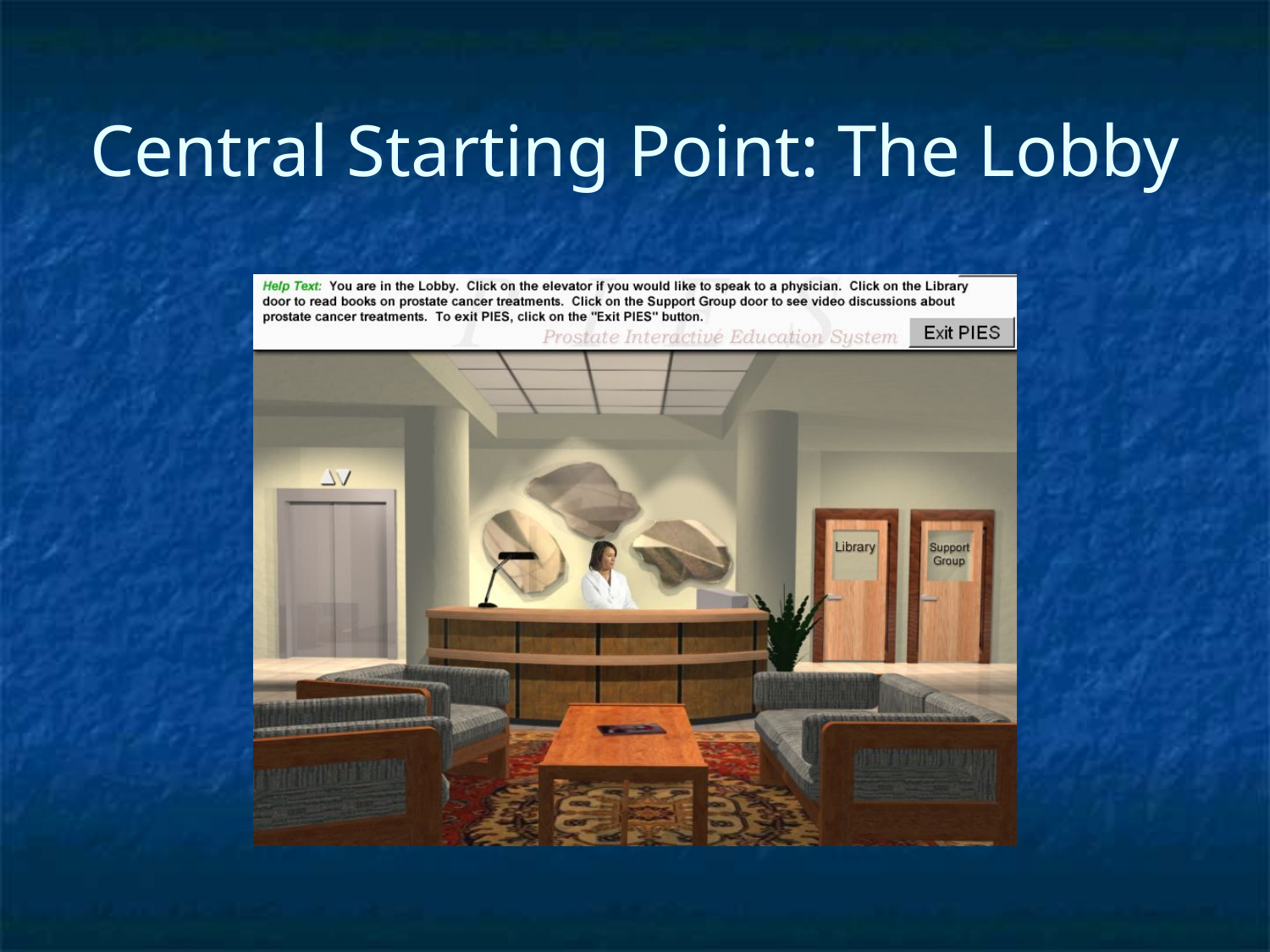

# Central Starting Point: The Lobby

## Slide 4
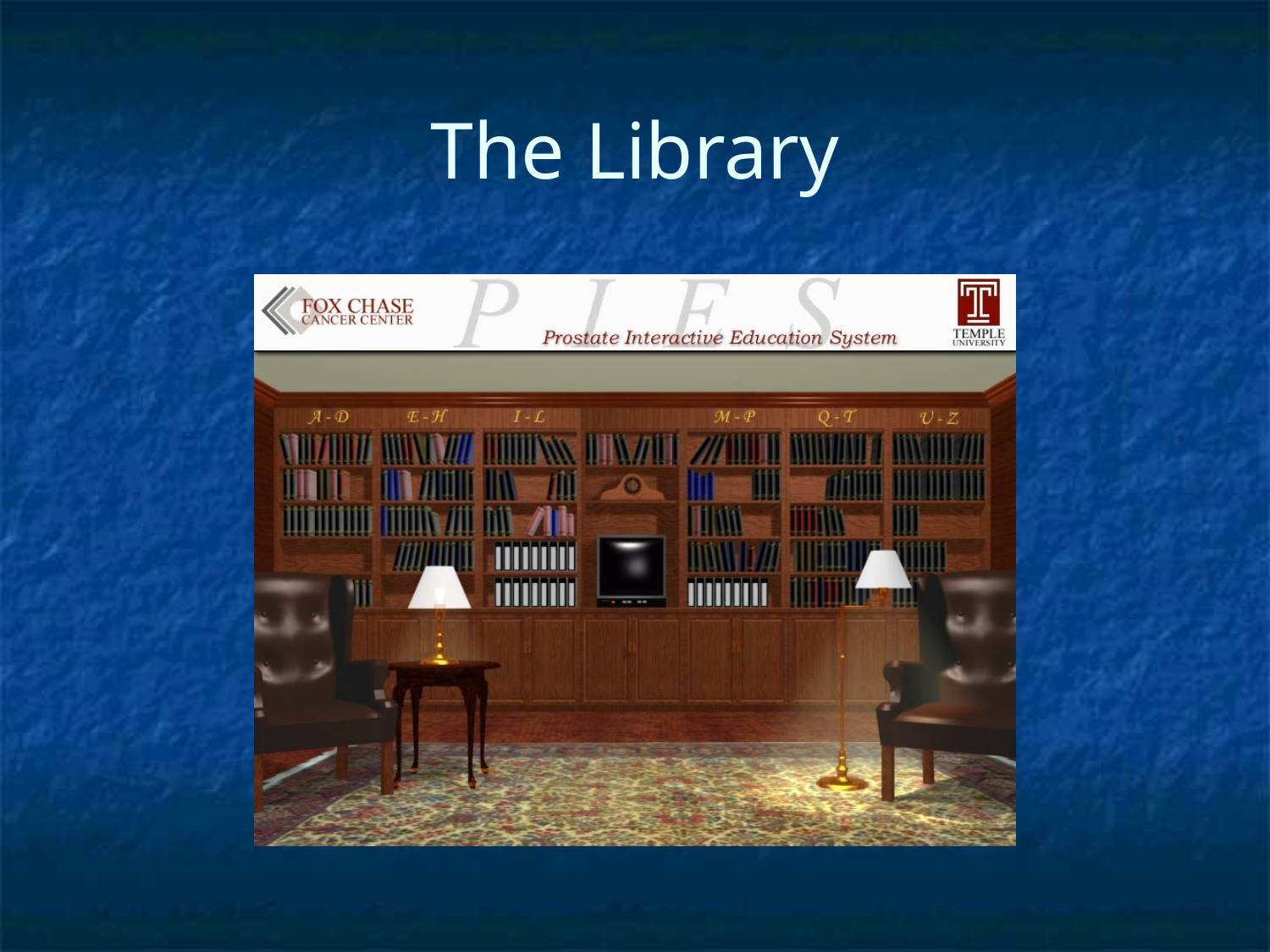

# The Library

## Slide 5
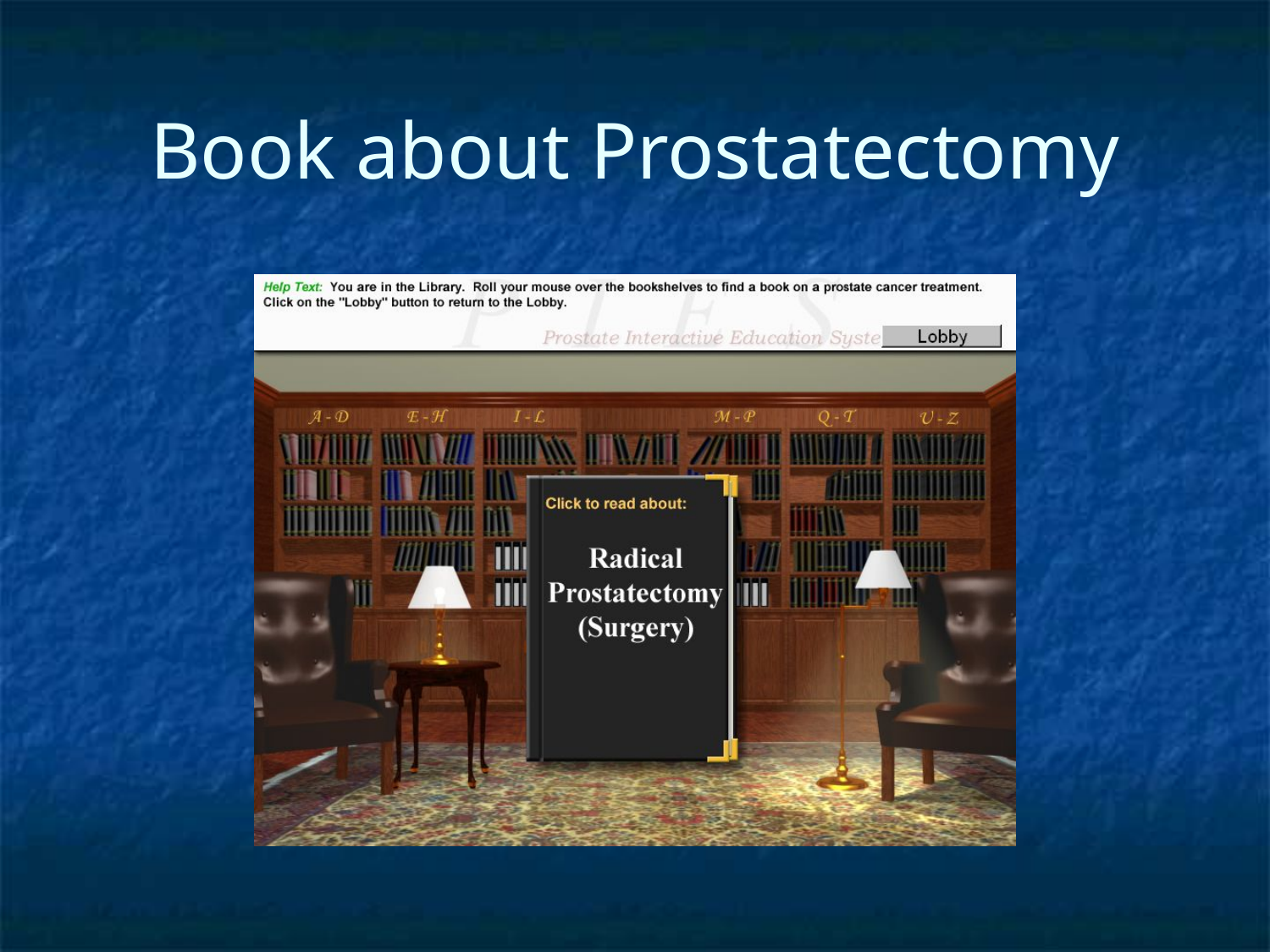

# Book about Prostatectomy

## Slide 6
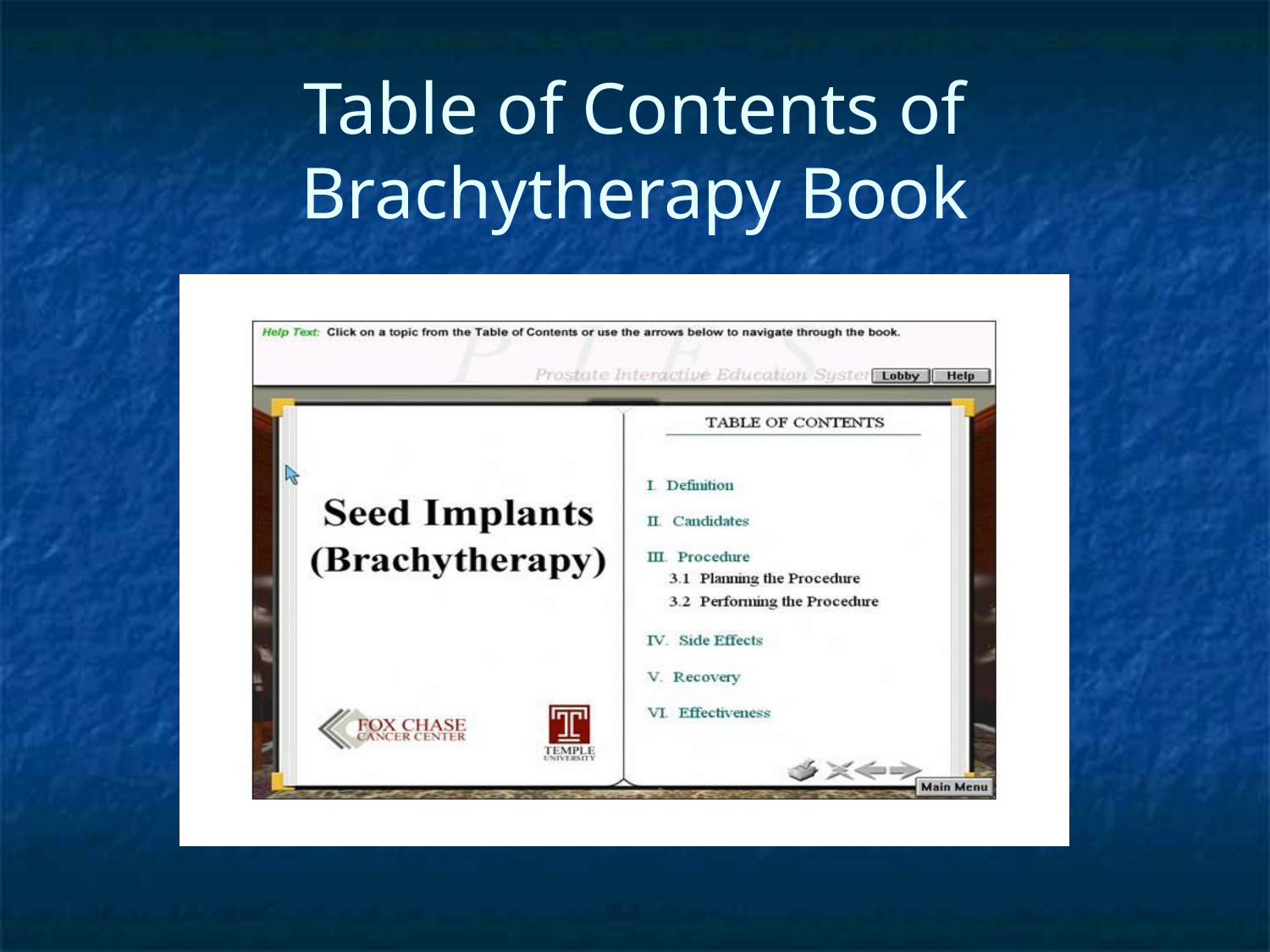

# Table of Contents of Brachytherapy Book

## Slide 7
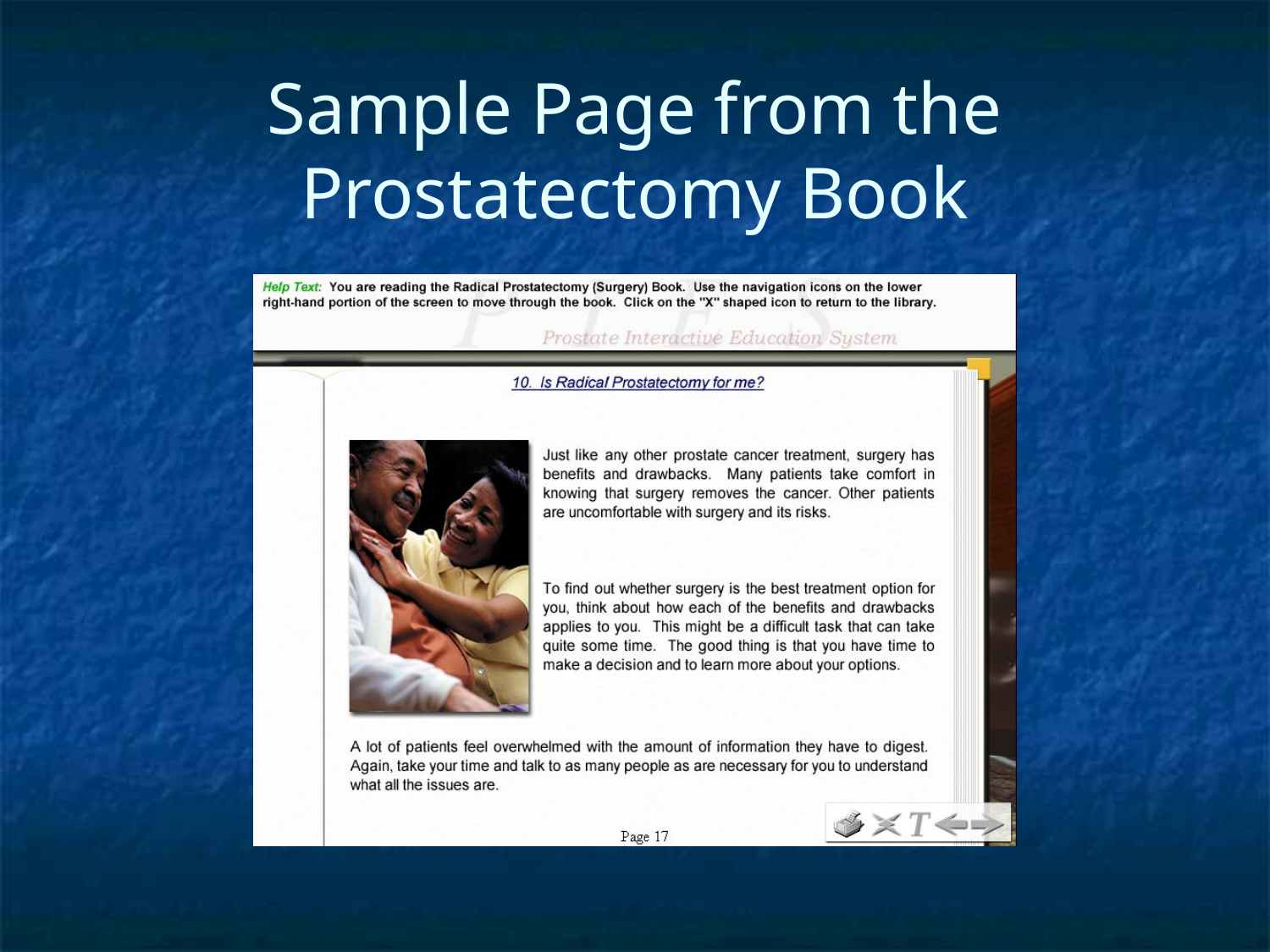

# Sample Page from the Prostatectomy Book

## Slide 8
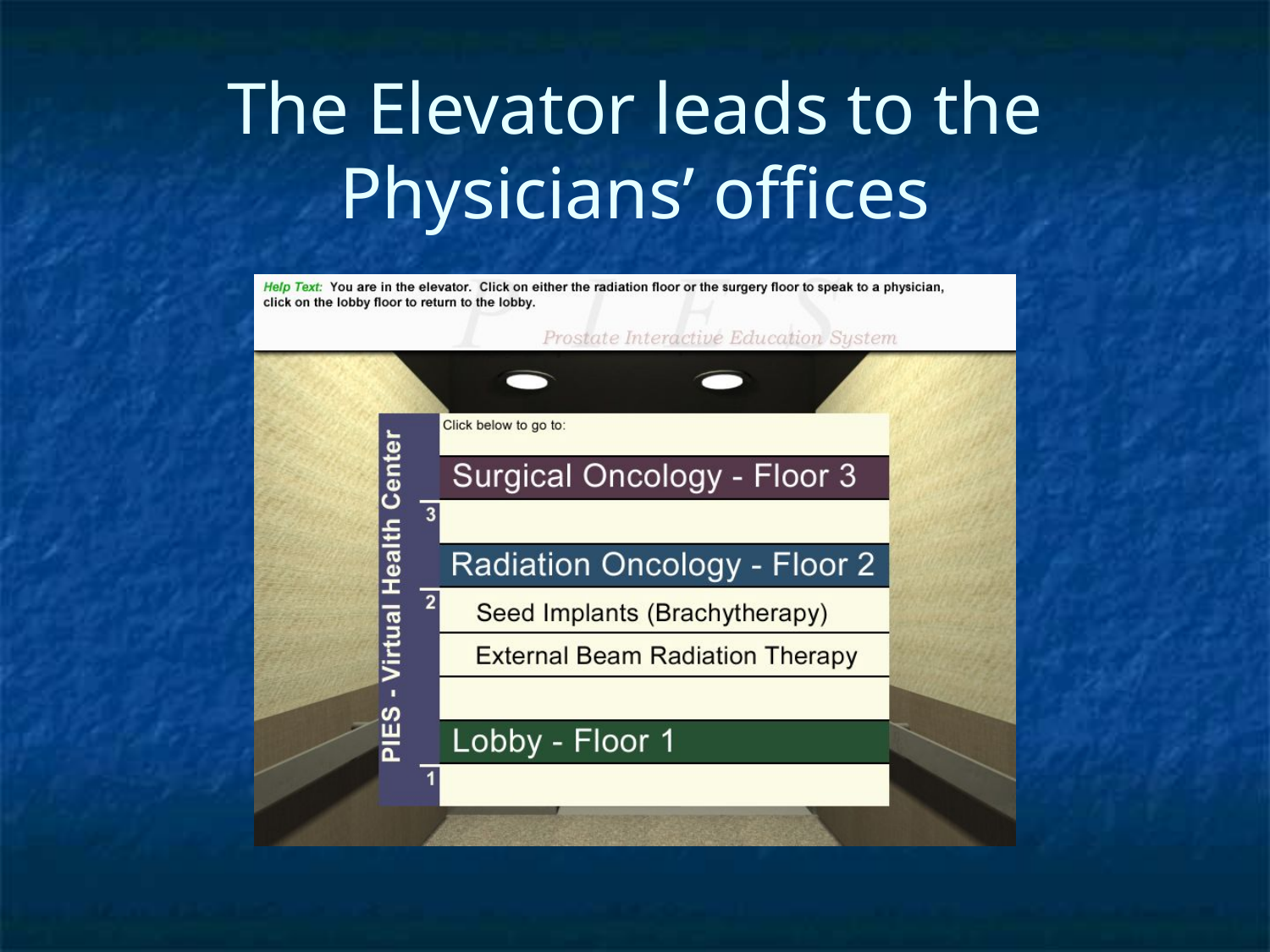

# The Elevator leads to the Physicians’ offices

## Slide 9
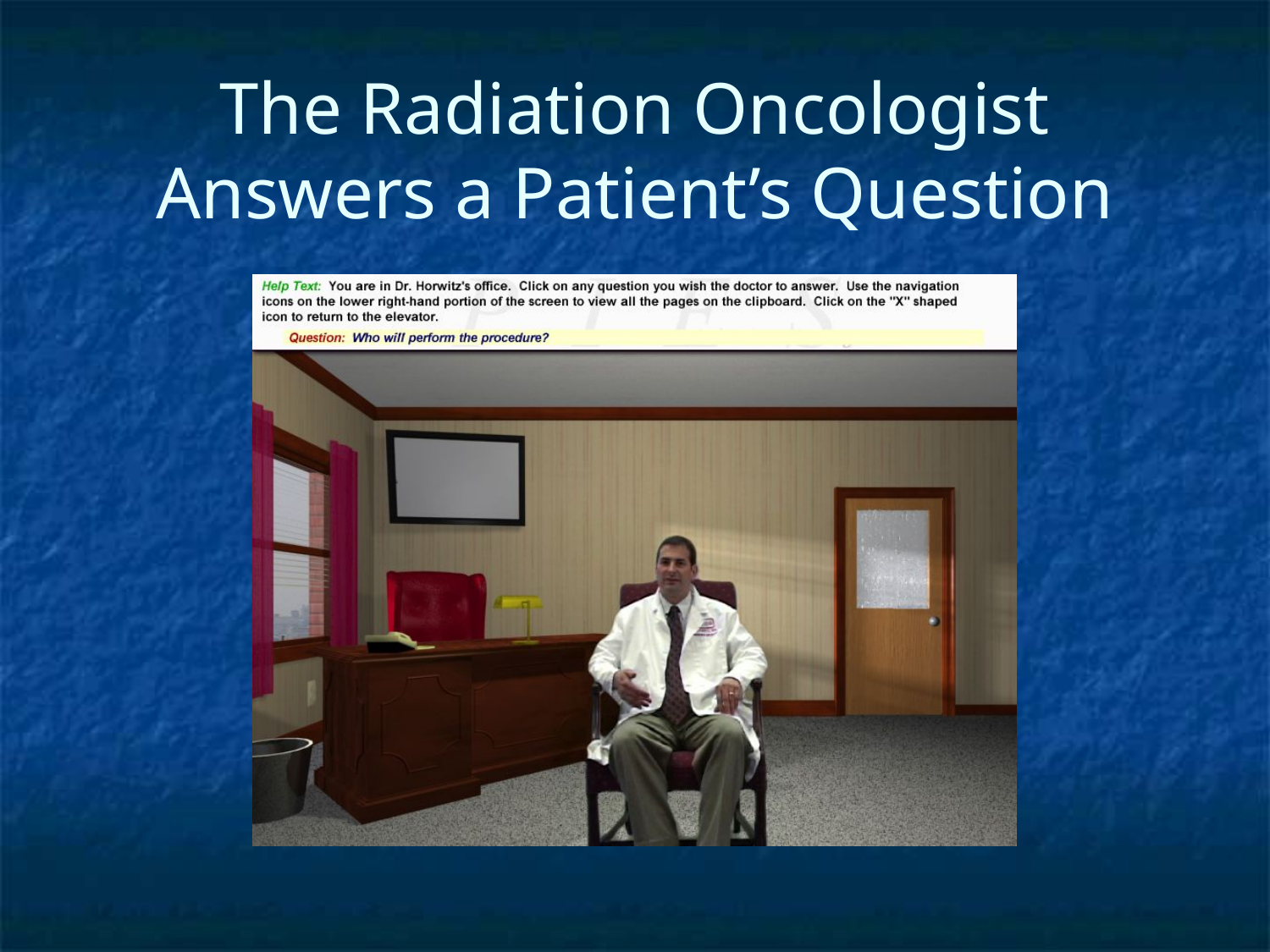

# The Radiation Oncologist Answers a Patient’s Question

## Slide 10
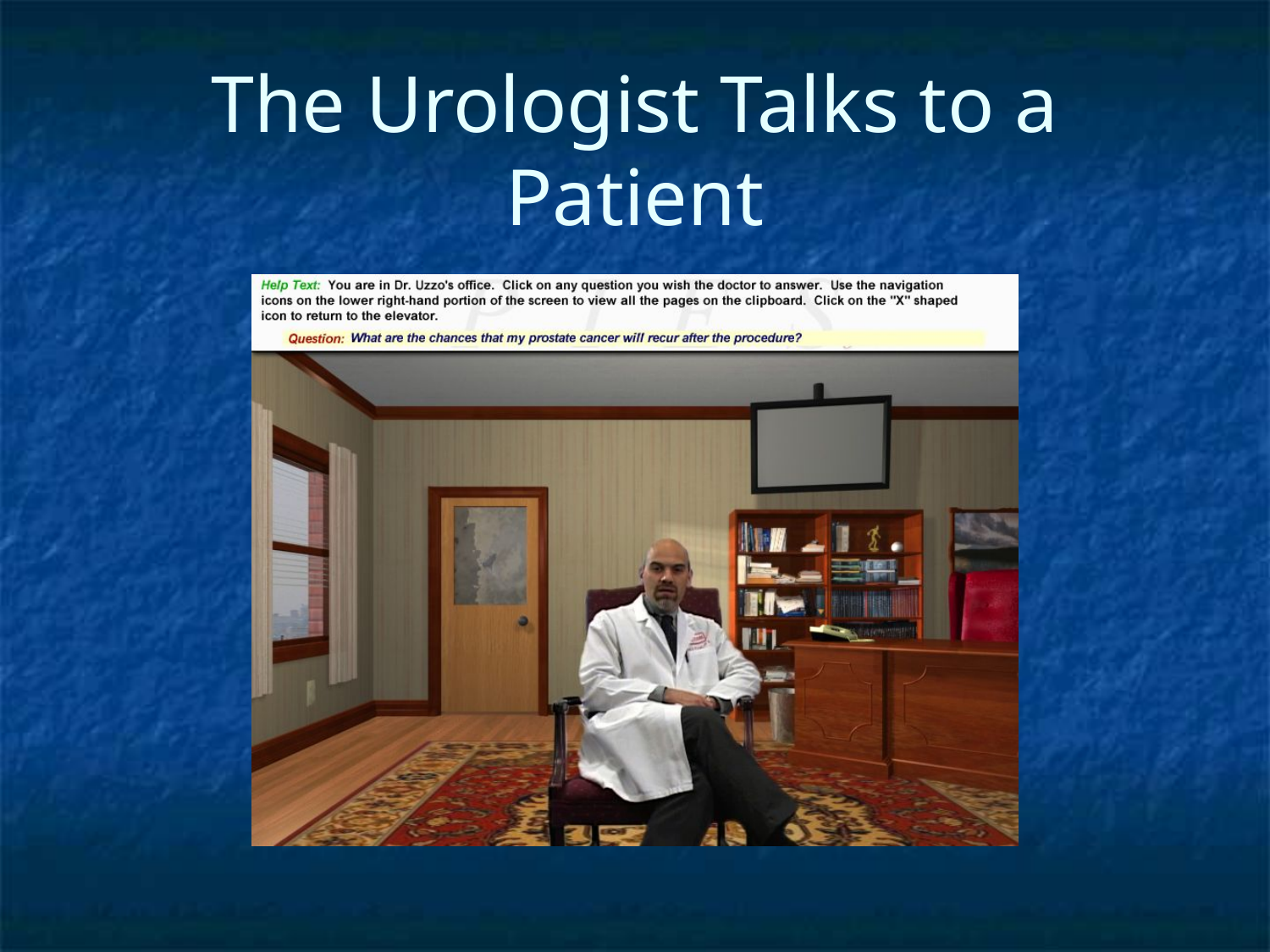

# The Urologist Talks to a Patient

## Slide 11
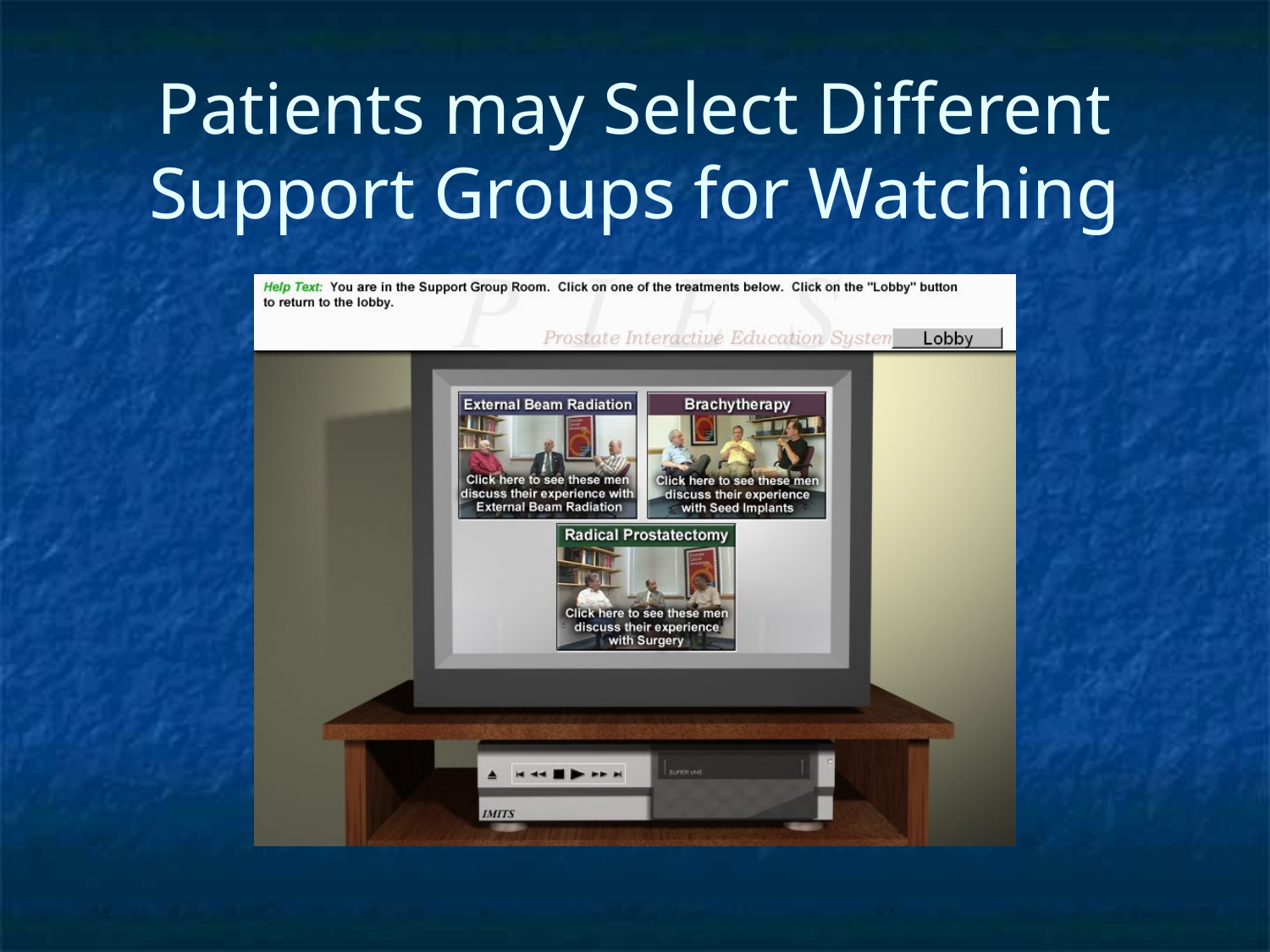

# Patients may Select Different Support Groups for Watching

## Slide 12
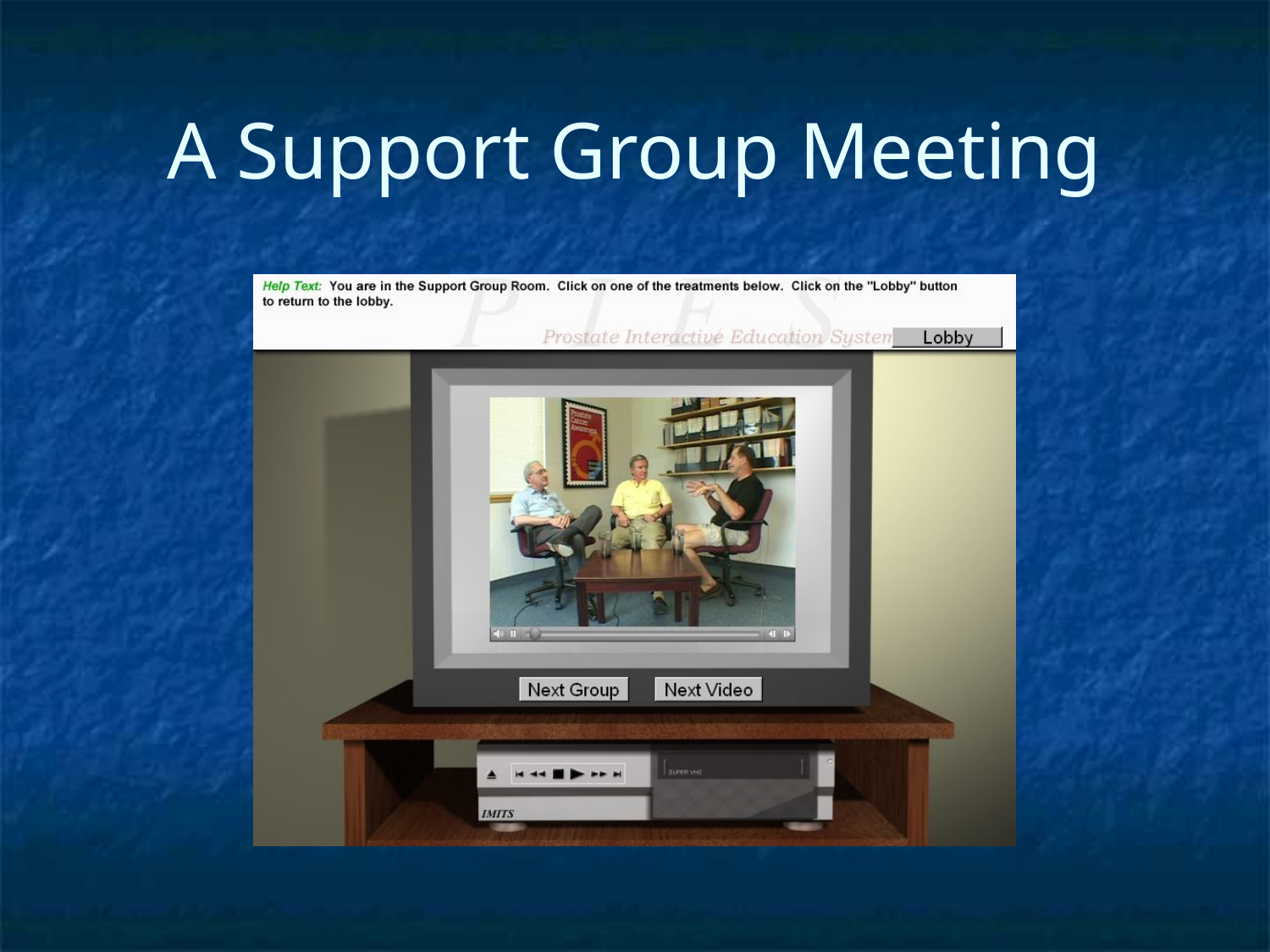

# A Support Group Meeting

## Slide 13
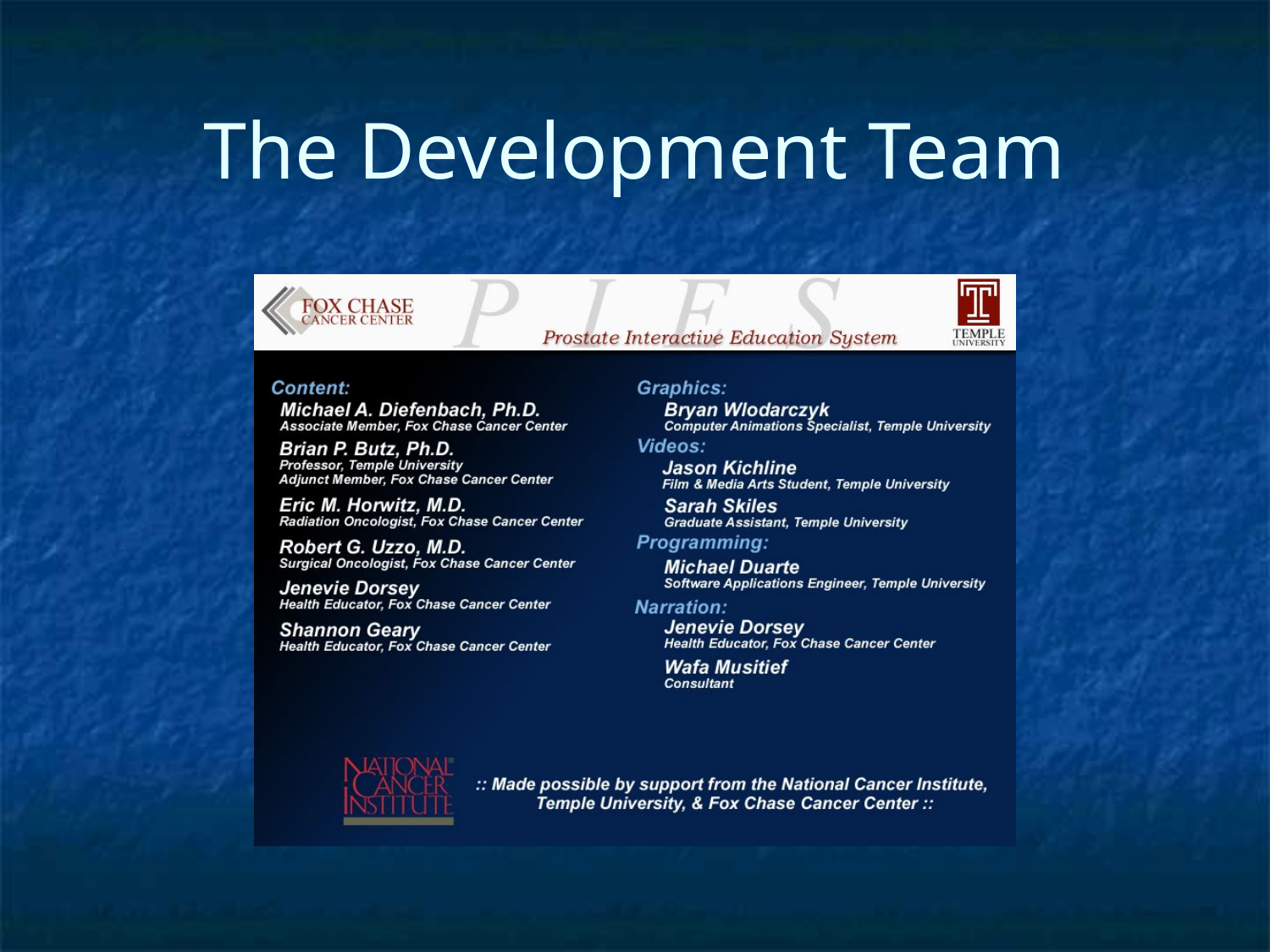

# The Development Team

## Slide 14
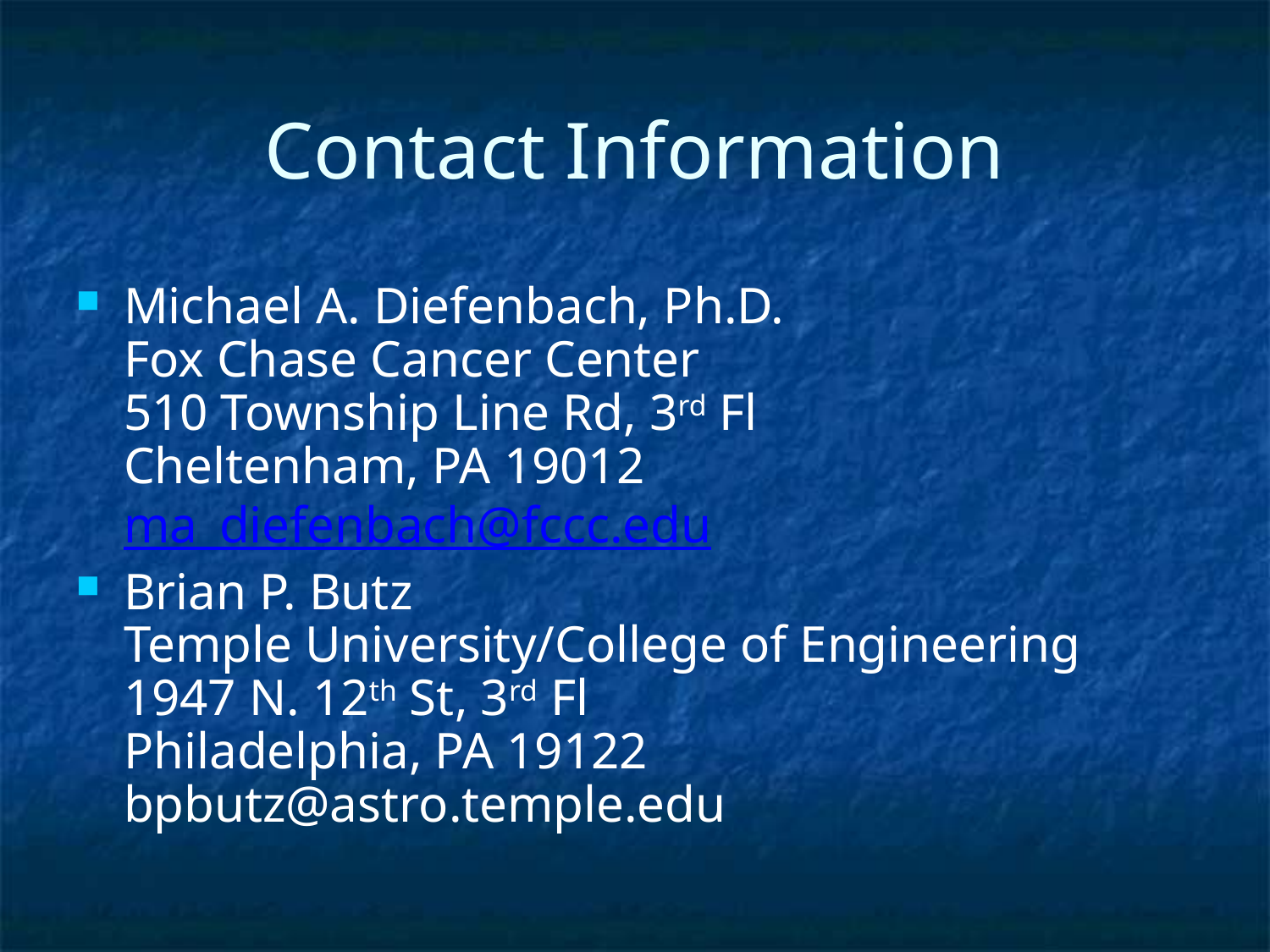

# Contact Information
Michael A. Diefenbach, Ph.D.
Fox Chase Cancer Center
510 Township Line Rd, 3rd Fl
Cheltenham, PA 19012
ma_diefenbach@fccc.edu
Brian P. Butz
Temple University/College of Engineering
1947 N. 12th St, 3rd Fl
Philadelphia, PA 19122
bpbutz@astro.temple.edu
